# Positive Airway Pressure, Mortality, and Cardiovascular Risk in Older Adults With Sleep Apnea

**DOI:** 10.1001/jamanetworkopen.2024.32468

**Published:** 2024-09-11

**Authors:** Diego R. Mazzotti, Lemuel R. Waitman, Jennifer Miller, Krishna M. Sundar, Nancy H. Stewart, David Gozal, Xing Song

**Affiliations:** 1 Division of Medical Informatics, Department of Internal Medicine, University of Kansas Medical Center, Kansas City; 2 Division of Medical Informatics, Department of Pulmonary, Critical Care and Sleep Medicine, University of Kansas Medical Center, Kansas City; 3 Department of Health Management and Informatics, School of Medicine, University of Missouri-Columbia; 4 College of Nursing, University of Nebraska Medical Center, Omaha; 5 Department Division of Pulmonary and Critical Care Medicine, Department of Medicine University of Utah, Salt Lake City; 6 Joan C. Edwards School of Medicine, Marshall University, Huntington, West Virginia

## Abstract

**Importance:**

Positive airway pressure (PAP) is the first-line treatment for obstructive sleep apnea (OSA), but evidence on its beneficial effect on major adverse cardiovascular events (MACE) and mortality prevention is limited.

**Objective:**

To determine whether PAP initiation and utilization are associated with lower mortality and incidence of MACE among older adults with OSA living in the central US.

**Design, Setting, and Participants:**

This retrospective clinical cohort study included Medicare beneficiaries with 2 or more distinct OSA claims identified from multistate, statewide, multiyear (2011-2020) Medicare fee-for-service claims data. Individuals were followed up until death or censoring on December 31, 2020. Analyses were performed between December 2021 and December 2023.

**Exposures:**

Evidence of PAP initiation and utilization based on PAP claims after OSA diagnosis.

**Main Outcomes and Measures:**

All-cause mortality and MACE, defined as a composite of myocardial infarction, heart failure, stroke, or coronary revascularization. Doubly robust Cox proportional hazards models with inverse probability of treatment weights were used to estimate treatment effect sizes controlling for sociodemographic and clinical factors.

**Results:**

Among 888 835 beneficiaries with OSA included in the analyses (median [IQR] age, 73 [69-78] years; 390 598 women [43.9%]; 8115 Asian [0.9%], 47 122 Black [5.3%], and 760 324 White [85.5%] participants; median [IQR] follow-up, 3.1 [1.5-5.1] years), those with evidence of PAP initiation (290 015 [32.6%]) had significantly lower all-cause mortality (hazard ratio [HR], 0.53; 95% CI, 0.52-0.54) and MACE incidence risk (HR, 0.90; 95% CI, 0.89-0.91). Higher quartiles (Q) of annual PAP claims were progressively associated with lower mortality (Q2 HR, 0.84; 95% CI, 0.81-0.87; Q3 HR, 0.76; 95% CI, 0.74-0.79; Q4 HR, 0.74; 95% CI, 0.72-0.77) and MACE incidence risk (Q2 HR, 0.92; 95% CI, 0.89-0.95; Q3 HR, 0.89; 95% CI, 0.86-0.91; Q4 HR, 0.87; 95% CI, 0.85-0.90).

**Conclusions and Relevance:**

In this cohort study of Medicare beneficiaries with OSA, PAP utilization was associated with lower all-cause mortality and MACE incidence. Results might inform trials assessing the importance of OSA therapy toward minimizing cardiovascular risk and mortality in older adults.

## Key Points

**Question:**

Is positive airway pressure therapy associated with lower mortality and incidence of major adverse cardiovascular events among Medicare beneficiaries with obstructive sleep apnea?

**Findings:**

In this cohort study of 888 835 older adults with obstructive sleep apnea in the central US, participants with evidence of positive airway pressure therapy initiation had significantly lower all-cause mortality and major adverse cardiovascular events incidence risk when compared with those without evidence of therapy.

**Meaning:**

These results might inform future trials assessing the importance of obstructive sleep apnea therapies toward minimizing cardiovascular risk and mortality in older adults.

## Teaser

This cohort study evaluates whether positive airway pressure use is associated with lower mortality and incidence of major adverse cardiovascular (CV) events among older adults with obstructive sleep apnea.

## Introduction

Obstructive sleep apnea (OSA) is highly prevalent (9%-37% in men and 4%-50% in women^1^), affecting nearly 1 billion people worldwide.^2^ OSA becomes more prevalent with age and obesity,^3^ and is associated with cardiovascular (CV) diseases and mortality.^4^ The CV risk due to OSA is greater among those with excessive sleepiness,^5^ worse nocturnal hypoxemia,^6^ and differential heart rate responses to respiratory events.^7^ Thus, there is increasing attention on therapies that may modify CV risk prevention through targeting OSA.

Positive airway pressure (PAP) is the first line of therapy for moderate to severe OSA. Despite epidemiological evidence suggesting OSA as a modifiable CV risk factor,^8,9^ randomized clinical trials (RCTs) failed to demonstrate that PAP prevents CV outcomes.^10^ Studies have shown that patient selection and treatment adherence may explain some of the negative results.^11^ Whether long-term PAP therapy prevents CV disease in a clinical population is a critical question that remains unanswered.

Insurance claims data allow the design of observational studies that complement RCTs when adopting causal inference methods. When robustly applied, they can help infer the effect of OSA therapies in more representative clinical settings.^12^ A study^13^ on a French nationwide claims database found that continuous PAP (CPAP) termination was associated with all-cause mortality and heart failure. In the US, Centers for Medicare and Medicaid Services (CMS) beneficiaries represent a large population of older adults with access to health care coverage. Studies exploring a sample of beneficiaries found that those with OSA had higher health care utilization when compared with matched controls.^14^ Moreover, those adherent to CPAP based on durable medical equipment claims had reduced risk of stroke^15^ and lower health care expenses among those with preexisting CV diseases.^16,17^ Although informative, studies on a sample of beneficiaries might not fully represent underserved regions in the US. Thus, the analysis of statewide Medicare claims might provide more generalizable effect estimates in these regions.

This study aimed to determine the association of PAP utilization with all-cause mortality and incidence of MACE and its components among Medicare beneficiaries in the central US by leveraging a robust analytical approach. Next, we established relevant PAP utilization groups based on first-year claims distribution and assessed their association with incident MACE and mortality. Finally, we provide estimates of the strength of associations stratified by relevant sociodemographic and clinical factors.

## Study Design and Methods

### Study Cohort and Design

A cohort of Medicare beneficiaries (aged >65 years) with 2 or more distinct OSA claims (see the eTable in Supplement 1) were identified from statewide, multiyear (2011-2020) Medicare fee-for-service claims data through the Greater Plains Collaborative Reusable Observable Unified Study Environment,^18^ with a catchment area across 11 states in the central US. The protocol was approved by institutional review boards at each participating institution. Informed consent was not required as only retrospective data were obtained and only deidentified data were made available to investigators. This study follows the Strengthening the Reporting of Observational Studies in Epidemiology (STROBE) reporting guideline for cohort studies. We required more than 1 year enrollment with Medicare before the first OSA claim, capturing newly diagnosed OSA and complete PAP utilization history. For the analysis of incident MACE, we further excluded beneficiaries with a history of MACE before OSA diagnosis.

We proposed 2 complementary designs estimating the average treatment effects of PAP initiation (eFigure 1 in Supplement 1) and PAP utilization exposure groups (eFigure 2 in Supplement 1). For the PAP initiation analyses, prescription time-distribution matching^19^ was used to identify time 0 (exposure assignment, covariate determination, and start of follow-up) for the group that did not initiate PAP, matched on the distribution of the time from diagnosis and start of PAP therapy in the group that initiated PAP. For the analysis of PAP utilization groups, we included beneficiaries who have not experienced events at the first anniversary of their PAP initiation date.

### PAP Utilization, Outcomes, and Covariates

Evidence of PAP initiation was based on the first initiation claim after OSA diagnosis. Because objective measures of adherence (eg, hours of PAP use) are not available in Medicare claims databases, our PAP utilization analysis was informed by the distribution of total PAP claims per patient at the end of the first year since PAP initiation (eFigure 3 in Supplement 1). PAP claim counts showed a bimodal distribution, suggesting 2 utilization patterns (a mode on 3 claims and a mode on 14 claims), suggestive of CMS PAP reimbursement models.^20^ We explored different PAP exposure group definitions to determine relevant cutoffs in the distribution of PAP claims (see eMethods in Supplement 1). A comparison of all methods informed the selection of the quartile (Q)-based PAP utilization definition (Q1, 1 to 7 claims; Q2, 8 to 12 claims; Q3, 13 to 15 claims; and Q4, >15 claims).

We assessed 2 primary outcomes: all-cause mortality and MACE, defined as a composite of first occurrence of myocardial infarction (MI), heart failure (HF), stroke, or coronary revascularization, identified by diagnostic and procedure codes (eTable in Supplement 1). Analyses using each MACE component were also performed. Covariates included age, sex, self-reported race (Asian, Black, American Indian, White, other, and unknown), socioeconomic status, history of type 2 diabetes, hypertension, obesity, atrial fibrillation, MACE (all-cause mortality models only), chronic obstructive pulmonary disease (COPD), chronic kidney disease (CKD), anxiety disorder, hypersomnia, insomnia, Charlson comorbidity index (CCI),^21^ prescriptions of anticoagulants, antihypertensives, antilipidemic agents, and blood glucose regulators (eTable in Supplement 1). Race was assessed due to known disparities in access to PAP therapy; it therefore represents a confounder. In addition, we provide stratified analyses by race to assess differences in estimates by race groups.

### Statistical Analysis

Sociodemographic and clinical characteristics were described between exposure groups using counts and percentages or medians and IQRs. Univariate associations between demographic and clinical history variables with PAP initiation exposure groups were performed using χ^2^ tests. Kaplan-Meier survival analyses and log-rank tests compared outcome survival-free curves between groups. We implemented a robust analytical approach to determine the association between PAP initiation or utilization on each outcome (eMethods in Supplement 1). Briefly, we derived propensity scores based on each exposure and used inverse probability of treatment weights in fully adjusted weighted Cox proportional hazards regression models to assess the association of exposures on outcomes. Results stratified by sociodemographic and clinical characteristics are also presented. Spline extrapolation analyses were used to represent hazard ratios (HR) as a function of total PAP claim counts during the first year. To determine whether a potential unmeasured confounder would nullify observed associations, we calculated E-values.^22^ Statistical significance was based on Bonferroni-corrected thresholds of *P* < .025 (2 primary outcomes). Data were analyzed from December 2021 to December 2023 using R version 4.3.0 (R Project for Statistical Computing).

## Results

### Sample Characterization

**Figure 1.**
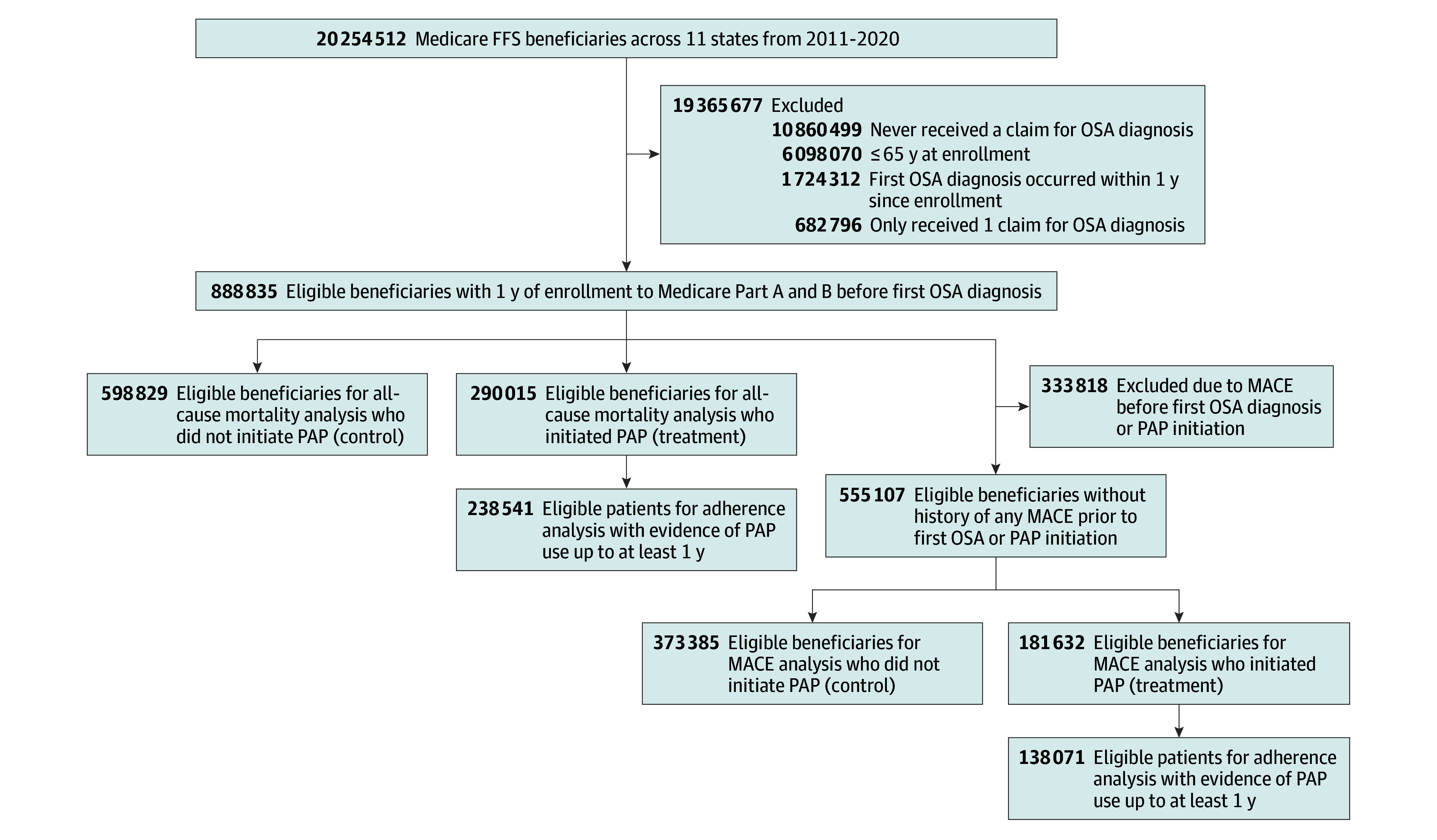
Study Cohort Definitions and Flowchart FFS indicates fee-for-service; MACE, major adverse cardiovascular events; OSA, obstructive sleep apnea; PAP, positive airway pressure.

Our sample included 888 835 Medicare beneficiaries with OSA (median [IQR] age, 73 [69-78] years; 390 598 women [43.9%]; 2365 American Indian [0.2%], 8115 Asian [0.9%], 47 122 Black [5.3%], 760 324 White [85.5%], 4061 other [0.5%], and 66 848 unknown race [7.5%] participants; median [IQR] follow-up, 3 [1.5-5.1] years). Figure 1 shows the study flowchart. Among participants, 290 015 (32.6%) had evidence of PAP initiation. Table 1 shows sample characteristics according to PAP initiation groups for the entire cohort, as well as incidence rates for all-cause mortality (among the entire cohort) and for MACE (among the cohort without prior MACE). Participants who had initiated PAP were younger, more likely to be women, White, and to have hypersomnia and insomnia, and less likely to have other comorbidities or use medications. The 5-year cumulative mortality rate was 20.5%. Among those included in the MACE incidence analysis (eg, no evidence of prior MACE at baseline; 555 017 individuals [62.4%]), the 5-year cumulative MACE incidence was 41.0%. Those who initiated PAP had a 5-year cumulative mortality rate and cumulative MACE incidence of 12.4% and 27.4%, respectively, vs 17.7% and 30.7% for those who did not initiate PAP.

**Table 1.** Sample Characteristics by Positive Airway Pressure (PAP) Initiation Groups Among Eligible Medicare Beneficiaries

Variable	Medicare beneficiaries by PAP initiation, No. (%)		*P* value^a^
	No evidence of PAP initiation (n = 598 820)	PAP initiation (n = 290 015)	
Age group, y			
65-69	159 589 (26.7)	89 414 (30.8)	<.001
70-74	189 168 (31.6)	94 955 (32.7)	
75-79	118 730 (19.8)	54 425 (18.8)	
≥80	131 333 (21.9)	51 221 (17.7)	
Sex			
Male	336 287 (56.2)	161 950 (55.8)	<.001
Female	262 533 (43.8)	128 065 (44.2)	
Race			
American Indian	1644 (0.3)	721 (0.2)	
Asian	5769 (1.0)	2346 (0.8)	<.001
Black	36 381 (6.1)	10 741 (3.7)	
White	502 414 (83.9)	257 910 (88.9)	
Other^b^	2765 (0.5)	1296 (0.4)	
Unknown	49 847 (8.3)	17 001 (5.9)	
Socioeconomic status			
Low-income subsidy or dual eligibility	177 559 (29.6)	72 957 (25.2)	<.001
Disease history^c^			
Hypersomnia	35 466 (5.9)	45 238 (15.6)	<.001
Insomnia	86 778 (14.5)	48 274 (16.7)	<.001
COPD	152 907 (25.5)	60 129 (20.7)	<.001
Type 2 diabetes	265 375 (44.3)	114 327 (39.4)	<.001
Hypertension	510 338 (85.2)	246 707 (85.1)	<.001
Obesity	261 592 (43.7)	111 381 (38.4)	<.001
Atrial fibrillation	97 427 (16.3)	34 943 (12.0)	<.001
Chronic kidney disease	150 949 (25.2)	55 058 (19.0)	<.001
Anxiety disorders	141 939 (23.7)	66 305 (22.9)	<.001
MACE	225 435 (37.6)	91 328 (31.5)	<.001
Myocardial infarction	81 770 (13.7)	31 329 (10.8)	<.001
Stroke	50 487 (8.4)	20 977 (7.2)	<.001
Heart failure	159 068 (26.6)	59 788 (20.6)	<.001
Coronary revascularization	36 154 (6.0)	18 324 (6.3)	<.001
Charlson Comorbidity Index, median (IQR)	3 (1-5)	2 (1-5)	<.001
Medication history^c^			
Any use of anticoagulants	88 130 (14.7)	36 141 (12.5)	<.001
Any use of antihypertensives	390 196 (65.2)	168 153 (58.0)	<.001
Any use of antilipemic agents	293 312 (49.0)	127 081 (43.8)	<.001
Any use of blood glucose regulators	143 556 (24.0)	52 235 (18.0)	<.001
Outcomes incidence			
All-cause mortality	105 768 (17.7)	35 981 (12.4)	<.001
MACE^d^^,^^e^	114 502 (30.7)	49 844 (27.4)	<.001
Myocardial infarction^e^	37 379(10.0)	15 607 (8.6)	<.001
Stroke^e^	30 817 (8.2)	14 485 (8.0)	.001
Heart failure^e^	79 293 (21.2)	33 260 (18.3)	<.001
Coronary revascularization^e^	16 644(4.5)	8526 (4.7)	<.001

Abbreviations: COPD, chronic obstructive pulmonary disease; MACE, major adverse cardiovascular events.

^a^ Calculated using χ^2^ tests.

^b^ The categories within the “other” race group were not available from the source Centers for Medicare and Medicaid Services data.

^c^ Positive history is defined when there is evidence of diagnosis codes indicating the condition before the first observed OSA diagnosis.

^d^ For MACE incidence, denominators represent patients without history of MACE.

^e^ For those with no evidence of PAP intitiation, the denominator is 373 385, and for those with PAP initiation, the denominator is 181 632.

### PAP Initiation, Mortality, and MACE

**Figure 2.**
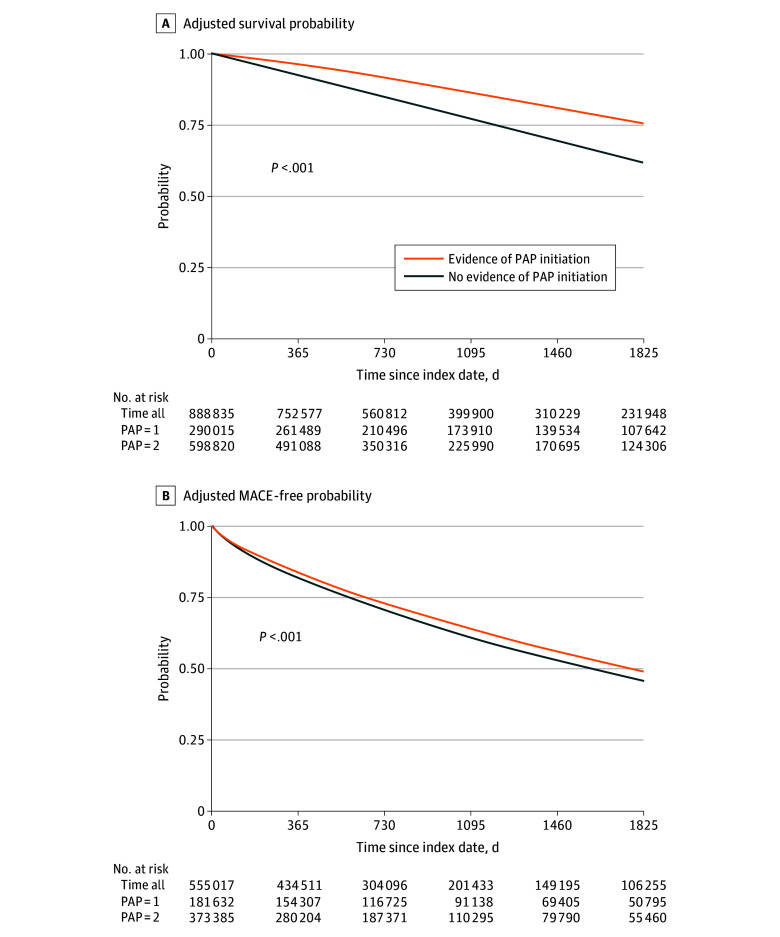
Adjusted Kaplan-Meier Survival Curves Describing the Survival Probabilities Between Positive Airway Pressure (PAP) Initiation Exposure Groups and All-Cause Mortality and Major Adverse Cardiovascular Events (MACE)

Kaplan-Meier survival curves for all-cause mortality and MACE according to PAP initiation groups are presented in Figure 2. Log-rank tests indicated significant differences in all-cause mortality and MACE-free survival probabilities (log-rank score for all-cause mortality, 247 722; log-rank score for MACE, 65 134; *P* < .001), with participants who had initiated PAP presenting greater survival.

Beneficiaries with evidence of PAP initiation had significantly lower all-cause mortality risk (HR, 0.53; 95% CI, 0.52-0.54) and lower MACE incidence risk (HR, 0.90; 95% CI, 0.89-0.91) when compared with those without (Table 2). The risk ratio of an unmeasured confounder (E-value) would need to be 2.47 or 1.36 to explain away these associations, respectively. Analyses of secondary outcomes (eFigure 3 in Supplement 1) indicated that patients with OSA with evidence of PAP initiation had significantly lower incidence risk of MI (HR, 0.84; 95% CI, 0.82-0.85; E-value, 1.48), HF (HR, 0.89; 95% CI, 0.88-0.90; E-value, 1.39), and stroke (HR, 0.86; 95% CI, 0.84-0.88; E-value, 1.41). Moreover, PAP initiation was associated with higher incidence risk of revascularization (HR, 1.08; 95% CI, 1.05-1.11; E-value, 1.37).

**Table 2.** Summary of Inverse Probability of Treatment Weight–Adjusted Cox Proportional Hazards Models Assessing the Association of Positive Airway Pressure (PAP) Initiation With All-Cause Mortality and Major Adverse Cardiovascular Events (MACE) in All Eligible Participants^a^

Evidence of PAP initiation	All-cause mortality, HR (95% CI)	MACE, HR (95% CI)
No	1 [Reference]	1 [Reference]
Yes	0.53 (0.52-0.54)	0.90 (0.89-0.91)

Abbreviation: HR, hazard ratio.

^a^ Results were derived from inverse probability of treatment weight–adjusted Cox proportional hazards models adjusted for age, sex, race, low-income subsidy or dual-eligibility indicator, type 2 diabetes, hypertension, obesity, atrial fibrillation, MACE (all-cause mortality only), chronic obstructive pulmonary disease, chronic kidney disease, anxiety disorder, hypersomnia, insomnia, Charlson comorbidity index, prescriptions of anticoagulants, antihypertensives, antilipidemic agents, and blood glucose regulators.

Stratified analyses (eFigure 4 in Supplement 1) revealed that the strength of association is consistent across subgroups with some differences. Greater protective associations of PAP initiation were observed among women (HR, 0.52; 95% CI, 0.51-0.53), those with obesity (HR, 0.51; 95% CI, 0.50-0.51), those with atrial fibrillation (HR, 0.47; 95% CI, 0.46-0.48), and those without hypersomnia (HR, 0.52; 95% CI, 0.52-0.53). Regarding MACE, we observed greater associations among those aged 75 years and older (75-79 years: HR, 0.89; 95% CI, 0.87-0.90; ≥80: HR, 0.86; 95% CI, 0.85-0.88), women (HR, 0.88; 95% CI, 0.87-0.89), those with lower socioconomic status (HR, 0.86; 95% CI, 0.85-0.87), those with insomnia (HR, 0.87; 95% CI, 0.85-0.89), obesity (HR, 0.88; 95% CI, 0.87-0.89), COPD (HR, 0.88; 95% CI, 0.86-0.89), type 2 diabetes (HR, 0.88; 95% CI, 0.87-0.89), atrial fibrillation (HR, 0.85; 95% CI, 0.83-0.87), and higher CCI scores (1-2 comorbidities: HR, 0.88; 95% CI, 0.87-0.90; 3-4 comorbidities: HR, 0.89; 95% CI, 0.88-0.91; ≥5 comorbidities: HR, 0.89; 95% CI, 0.87-0.91).

Stratified analyses regarding secondary outcomes showed similar results (eFigure 5 in Supplement 1), with important differences. Regarding MI, the greatest PAP initiation associations were observed among women (HR, 0.80; 95% CI, 0.79-0.82) and those with lower socioeconomic status (HR, 0.80; 95% CI, 0.78-0.82), hypersomnia (HR, 0.77; 95% CI, 0.73-0.81), insomnia (HR, 0.79; 95% CI, 0.76-0.82), and anxiety disorders (HR, 0.79; 95% CI, 0.76-0.82). Regarding HF, the greatest associations were observed in those aged 75 years and older (75-79 years: HR, 0.86; 95% CI, 0.84-0.88; ≥80 years: HR, 0.86; 95% CI, 0.84-0.88), and those with atrial fibrillation (HR, 0.84; 95% CI, 0.81-0.87). Regarding stroke, the greatest associations were observed among women (HR, 0.83; 95% CI, 0.81-0.85) and those with lower socioeconomic status (HR, 0.82; 95% CI, 0.80-0.84), obesity (HR, 0.82; 95% CI, 0.80-0.84), COPD (HR, 0.81; 95% CI, 0.79-0.84), hypertension (HR, 0.85; 95% CI, 0.83-0.86), and a CCI score of 5 more (HR, 0.82; 95% CI, 0.79-0.84). Regarding coronary revascularization, the greatest detrimental associations of PAP initiation were observed among those aged older than 80 years (HR, 1.18; 95% CI, 1.12-1.24), men (HR, 1.10; 95% CI, 1.08-1.13), and those taking antihypertensives (HR, 1.12; 95% CI, 1.09-1.15).

### PAP Utilization, Mortality, and MACE

Exploratory analyses informed that a Q-based definition of PAP utilization based on first year claim counts (Q1, 1-7; Q2, 8-12; Q3, 13-15; and Q4, >15 claims) provided a realistic representation of utilization patterns with clinically relevant variation in incident outcome risk (eFigure 6, eFigure7, eFigure 8, and eFigure 9 in Supplement 1). Among patients with evidence of initiating PAP, higher Qs (ie, higher PAP utilization) were progressively associated with lower all-cause mortality (Q2 HR, 0.84; 95% CI, 0.81-0.87; Q3 HR, 0.76; 95% CI, 0.74-0.79; Q4 HR, 0.74; 95% CI, 0.72-0.77) and lower MACE incidence (Q2 HR, 0.92; 95% CI, 0.89-0.95; Q3 HR, 0.89; 95% CI, 0.86-0.91; Q4 HR, 0.87; 95% CI, 0.85-0.90), when compared with those with 7 or more PAP claims (Table 3). Similar results were observed for secondary outcomes (eFigure 10 in Supplement 1). No evidence of association with incident coronary revascularization was observed.

**Table 3.** Summary of Inverse Probability of Treatment Weight–Adjusted Cox Proportional Hazards Models Assessing the Association of Positive Airway Pressure (PAP) Utilization Exposure Groups Based on Quartiles (Q) of PAP Claim Counts With All-Cause Mortality and Major Adverse Cardiovascular Events (MACE) Among All Participants^a^

PAP utilization based on claims	All-cause mortality, HR (95% CI)	MACE, HR (95% CI)
Q1 (1-7 claims)	1 [Reference]	1 [Reference]
Q2 (8-12 claims)	0.84 (0.81-0.87)	0.92 (0.89-0.95)
Q3 (13-15 claims)	0.76 (0.74-0.79)	0.89 (0.86-0.91)
Q4 (>15 claims)	0.74 (0.72-0.77)	0.87 (0.85-0.90)

Abbreviation: HR, hazard ratio.

^a^ Results were derived from inverse probability of treatment weight–adjusted Cox proportional hazards models adjusted for age, sex, race, low-income subsidy or dual-eligibility indicator, type 2 diabetes, hypertension, obesity, atrial fibrillation, MACE (all-cause mortality only), chronic obstructive pulmonary disease, chronic kidney disease, anxiety disorder, hypersomnia, insomnia, Charlson comorbidity index, prescriptions of anticoagulants, antihypertensives, antilipidemic agents, and blood glucose regulators.

Analyses stratified by sociodemographic and clinical characteristics (eFigure 11 in Supplement 1) were consistent overall, supporting protective associations of higher PAP utilization against primary outcomes. Regarding mortality, differences include greater protective associations among those aged 65 to 69 years and without hypertension. Regarding MACE, greater protective associations were observed among those aged 65 to 69 years, higher socioeconomic status, and those without hypertension, atrial fibrillation, and insomnia. Regarding secondary outcomes (eFigure 12 in Supplement 1), greater protective associations were observed against MI among those aged 80 or more years, those with lower socioeconomic status, and those with hypersomnia. Regarding HF, greater protective associations were observed among those aged 65 to 69 years, those without insomnia or hypertension, and those with lower CCI. Regarding stroke, greater protective associations were observed among those without insomnia. No consistent associations were observed regarding coronary revascularization.

## Discussion

This cohort study provided a systematic assessment of associations of PAP initiation and utilization with mortality and MACE among Medicare beneficiaries in the central US. Findings suggest significant associations between PAP initiation and utilization and lower all-cause mortality and MACE incidence. Associations were also significant for MACE components with consistent effects on MI, HF, and stroke. Higher PAP utilization was progressively associated with lower incidence of outcomes, with consistent results in stratified analysis. PAP initiation was also associated with higher incidence risk of revascularization, particularly among the oldest patients, men, and those taking antihypertensive medications.

Extensive epidemiological evidence suggests that moderate to severe OSA is associated with stroke,^23^ atrial fibrillation^24^ MI,^25^ and CV mortality.^26^ Mechanisms explaining this relationship include sympathetic activation, endothelial dysfunction, oxidative stress, systemic inflammation, cardiac remodeling due to hypoxemia, and intrathoracic pressure swings.^27^ RCTs have established that PAP improves daytime symptoms, mood, and quality of life,^28,29^ and reduces systolic and diastolic blood pressure,^30^ especially in resistant hypertension.^31^ PAP has positive effects on long-term survival in patients with ischemic stroke and moderate to severe OSA,^32^ and it has been associated with lower rates of CV events over 10 years.^25^ However, recent RCTs investigating the effect of CPAP on CV events were negative,^10^ although post-hoc on-treatment analyses suggested protective effects of CPAP adherence and reduced MACE risk.^10^ Studies have suggested that patient selection and treatment adherence may partially explain differences between RCTs and observational studies.^11^ Less than 20% of patients seeking care in sleep clinics would be eligible for these RCTs.^12^ Many patients present with excessive daytime sleepiness, which was an important ethical exclusion criterion in RCTs.^11^ However, this subgroup is the one at increased risk for incident CV diseases.^5,33,34^

These observations suggest that alternative approaches using causal inference in well-designed observational studies could provide more generalizable evidence toward the role of PAP therapy on CV risk. Exploring clinical data using robust methods that minimize the biases and confounding inherent to observational studies may help inform timely and cost-effective evidence generation. This has been demonstrated by the current investigation, as it provided relevant and generalizable evidence about estimated effects of PAP initiation and utilization among older adults. In the context of a recently published report from the Agency for Healthcare Research and Quality suggesting lack of strong evidence of the role of PAP on long-term outcomes,^35^ such studies are fundamental to fill this evidence gap.

Our study has identified important associations within subgroups. We observed greater beneficial associations of PAP initiation against MACE among those 75 years and older, suggesting the importance of primary CV prevention at older ages. However, results conflict with a 3-month RCT of CPAP therapy among adults older than 70 years with moderate to severe OSA, which did not find significant effects on blood pressure, although significant improvements in sleepiness were observed.^36^ Analysis among those older than 80 years did not find significant effects on sleepiness.^37^ Our study found greater beneficial associations of PAP utilization in those who were 65 to 69 years. Differences may be explained by length of follow-up and study design considerations. We also observed greater PAP associations with mortality and MACE among women, particularly following menopause, in agreement with prior studies.^28^ It is likely that women are subjected to stronger acute detrimental effects of OSA following menopause, and therefore might experience stronger therapeutic effects. Women with OSA are also more likely to have comorbid metabolic conditions.^38^ However, little is known about sex differences in PAP treatment responses,^39^ particularly at older ages. Patients with lower socioeconomic status may be less receptive to PAP therapy^40^ and report poorer treatment adherence.^41^ Yet, our results demonstrate that those with lower socioeconomic status may have greater benefit of PAP against MACE. These results highlight opportunities to implement programs designed to minimize disparities.^42^ Our study also identified greater associations of PAP initiation among those with comorbid conditions, particularly obesity, atrial fibrillation, COPD, and type 2 diabetes. It is likely that these subgroups are at a greater underlying risk with the added comorbid impact,^4,43^ and therefore therapy benefit might be greater. Importantly, subgroups with evidence for comorbidities might also represent patients at highest risk for early termination of CPAP,^44^ which suggests that efforts to support therapy continuation might benefit these patients.

Greater associations of PAP initiation against MACE were observed among those with evidence of hypersomnia, due to greater effects against MI. Results were corroborated by greater effects of greater PAP utilization against MI among those with hypersomnia. These results support the well-established epidemiological relationship between excessive sleepiness and increased CV risk among those with OSA.^5,33,34^ Importantly, granular measures of excessive sleepiness might provide a more robust phenotype when compared with claims for hypersomnia. Moreover, only approximately 9% of our study cohort had evidence of hypersomnia, which suggests it might not capture all patients with this phenotype. We also observed greater effects of PAP initiation against MACE among those with evidence of insomnia. These results support studies that demonstrate greater CV risk associated with comorbid insomnia and sleep apnea,^45^ and might help guide future RCTs assessing the role of strategies to increase PAP adherence among higher-risk subgroups. Associations between PAP initiation and increased risk of coronary revascularization procedures were observed, suggesting potentially damaging effects of PAP. Interestingly, these were mostly concentrated in those 80 years and older, and analysis of PAP utilization was not significant. An alternative explanation might relate to the fact that those who initiated PAP were also more likely to receive other relevant CV procedures. Future studies assessing the role of PAP on revascularization procedures in older adults might provide further insights.

### Limitations

Our study has important limitations. First, our cohort identification approach was based on diagnostic codes, preventing characterization of disease severity based on physiological traits.^6,7^ However, we used a validated algorithm with excellent predictive performance across multiple sites,^46^ suggesting that we reliably captured individuals with OSA. Second, claims-based PAP utilization definitions may not accurately represent objective definitions of PAP use. However, hours of use (often used as the only exposure in telemonitoring studies) is not the only determinant of PAP efficacy. Factors such as sleep time on efficacious PAP relative to total sleep time, optimal pressure, minimal leak, minimal residual apnea-hypopnea index, and timing of PAP utilization during the night^47^ might be important predictors of successful therapy. We encourage PAP vendors to expand academic-industry partnerships toward facilitating privacy-preserving linkage of PAP telemonitoring data with granular information from medical records (eg, whether PAP was prescribed but not initiated), claims, patient reported outcomes, and wearables, as they will be fundamental for studies aimed to determining effects of PAP therapy on short- and long-term outcomes. Third, while we included relevant covariates, there was lack of granular information about body mass index, blood pressure, and laboratory measurements. Other factors such as diet, tobacco history, physical activity, and healthy adherer behaviors^48^ were unavailable and could explain some observed effects. Individuals who have decided to initiate and continue to use PAP may be more likely to adhere to healthier behaviors that may not have been captured in this cohort. Nevertheless, we provided E-values as a guide to contextualize the associations of potential unmeasured confounders on our reported associations. For reference, established 5-year mortality risk factors among older adults observed in the Cardiovascular Health Study had lesser associations than our reported E-values, suggesting that even in the presence of unmeasured confounding, our reported associations on PAP initiation are expected to be significant.^49^ Information about other OSA treatments, particularly among those without evidence of PAP initiation, was not available and may impact our observed associations. Additionally, our study focuses on Medicare beneficiaries in the central US, and extrapolation to other demographics cannot be made. Information about ethnicity was not accurately represented in CMS and therefore stratified analyses by ethnicity could not be performed. Studies in midlife adults would provide important insights about OSA natural history and early CV prevention. However, our study may generalize to a very large proportion of older adults, as more than 95% of those older than 65 years in the US are enrolled in Medicare.

## Conclusions

In this cohort study of Medicare beneficiaries with OSA, PAP utilization based on claims was associated with lower mortality and MACE incidence. Results support that PAP may have beneficial effects against mortality and CV diseases. This study has the potential to inform future trials assessing the importance of OSA therapy initiation and maintenance toward minimizing adverse health outcomes leading to healthier lives. These results may also help to inform more personalized strategies to improve PAP adherence and efficacy among older adults.

## Notes

Supplement 1.
eMethods.
**eTable. **Definitions of Computable Phenotypes Used for Study Exposure, Outcomes, and Covariates **eFigure 1. **Overview of the PAP Initiation Analysis Study Design **eFigure 2. **Overview of the PAP Utilization Exposure Group Study Design **eFigure 3. **Summary of IPTW-Adjusted Cox Proportional Hazards Model Assessing the Effect of PAP Initiation on Myocardial Infarction (MI), Heart Failure (HF), Stroke (STROKE) and Coronary Revascularization (REVASC) in all Eligible Participants **eFigure 4. **Summary of Inverse Probability Of Treatment Weights (IPTW)–Adjusted Cox Proportional Hazards Models Assessing the Effect of PAP Initiation on All-Cause Mortality (ACM) and MACE Within Categories of Relevant Sociodemographic and Clinical Characteristics **eFigure 5. **Summary of Inverse Probability Of Treatment Weights (IPTW)–Adjusted Cox Proportional Hazards Models Assessing the Effect of PAP Initiation on Myocardial Infarction (MI), Heart Failure (HF), Stroke (STROKE) and Coronary Revascularization (REVASC) Within Categories of Relevant Sociodemographic and Clinical Characteristics **eFigure 6. **Distribution of Total Count of Positive Airway Pressure (PAP) Claims During the First Year of PAP Utilization **eFigure 7. **Categorization of Positive Airway Pressure (PAP) Claim Counts Distribution First Year of PAP Utilization According to Quantile-Based Definitions (Quantile_Median, Quantile_Tertile, Quantile_Quartile), Rule-Based Definitions (Rule_Based), Equal Spaced (EqualSpaced_By4, EqualSpaced_By8) and Based on an Empirical Method That Identified the Most Discriminant Cut Point For All-Cause Mortality and MACE **eFigure 8. **Summary of Inverse Probability Of Treatment Weights (IPTW)–Adjusted Cox Proportional Hazards Models Assessing the Effect of Different PAP Exposure Group Definitions at First Year of PAP Utilization on Primary Outcomes: All-Cause Mortality (ACM) and Major Adverse Cardiovascular Events **eFigure 9. **Spline Extrapolation Analyses Representing Estimated Hazards Ratio (HR) as a Function of the Total Positive Airway Pressure (PAP) Claim Counts During First Year of PAP Utilization for Each Primary and Secondary Study Outcome **eFigure 10. **Summary of IPTW-Adjusted Cox Proportional Hazards Models Assessing the Effect of PAP Utilization Exposure Groups Assessing the Effect of PAP Utilization Exposure Groups Based on Quartiles of PAP Claim Counts on Myocardial Infarction (MI), Heart Failure (HF), Stroke (STROKE) and Coronary Revascularization (REVASC) in All Eligible Participant **eFigure 11. **Summary of Inverse Probability of Treatment Weights (IPTW)–Adjusted Cox Proportional Hazards Models Assessing the Effect of PAP Exposure Groups Based on Quartiles of PAP Utilization During First Year on All-Cause Mortality (ACM) and MACE Within Categories of Relevant Sociodemographic and Clinical Characteristics **eFigure 12. **Summary of Inverse Probability Of Treatment Weights (IPTW)–Adjusted Cox Proportional Hazards Models Assessing the Effect of PAP Exposure Groups Based on Quartiles of PAP Utilization During First Year on Myocardial Infarction (MI), Heart Failure (HF), Stroke (STROKE) and Coronary Revascularization (REVASC) Within Categories of Relevant Sociodemographic and Clinical Characteristics
eReferences

Supplement 2.
Greater Plains Collaborative Team Members

Supplement 3.
Data Sharing Statement
