# Supplementary material for: Positive Airway Pressure, Mortality, and Cardiovascular Risk in Older Adults With Sleep Apnea
Source: JAMA Netw Open. 2024 Sep 11;7(9):e2432468. doi: 10.1001/jamanetworkopen.2024.32468 (PMC11391331; doi:10.1001/jamanetworkopen.2024.32468)
Supplement: Supplement 2. — Greater Plains Collaborative Team Members [file jamanetwopen-e2432468-s002.pdf]

Supplemental Online Content: Nonauthor Collaborators
*First name, last name, and suffix (if applicable) are required and will appear in PubMed.

***Group Name(s):**

***First Name and Middle Initial(s)**	***Last Name**	***Suffix (eg, Jr, III)**	Academic Degrees	Institution	Location (city, state/province, country)	Role or Contribution, eg, chair, principal investigator	Group (if more than 1 Group listed in the byline) and/or Subgroup (eg, Steering Committee)
Sravani	Chandaka						
Kelechi (KayCee)	Anuforo						
Lav	Patel						
Daryl	Budine						
Nathan	Hensel						
Siddharth	Satyakam						
Sharla	Smith						
Dennis	Ridenour						
Cheryl	Jernigan						
Carol	Early						
Kyle	Stephens						
Kathy	Jurius						
Abbey	Sidebottom						
Cassandra	Rodgers						
Hong	Zhong						
Vino	Raj						
Victor	Melendez						
Angie	Hare						
Roman	Melamed						
Curtis	Anderson						
Thomas	Schouweile						
Christine	Roering						
Philip	Payne						
Snehil	Gupta						
John	Newland						
Albert	Lai						
Joyce	Balls-Berry						
Janine	Parham						
Evin	Fritschle						
Shanelle	Cripps						
Kirk	Knowlton						
Channing	Hansen						

Supplemental Online Content: Nonauthor Collaborators
*First name, last name, and suffix (if applicable) are required and will appear in PubMed.

***First Name and Middle Initial(s)**	***Last Name**	***Suffix (eg, Jr, III)**	Academic Degrees	Institution	Location (city, state/province, country)	Role or Contribution, eg, chair, principal investigator	Group (if more than 1 Group listed in the byline) and/or Subgroup (eg, Steering Committee)
Erna	Serezlic						
Benjamin	Horne						
Jeff	VanWormer						
Judith	Hase						
Janet	Southworth						
Eric	Larose						
Mary	Davis						
Laurel	Hoeth						
Sandy	Strey						
Brad	Taylor						
Kris	Osinski						
April	Haverty						
Alex	Stoddard						
Sarah	Cornell						
Phoenix	Do						
Lucy	Bailey						
Beth	McDonough						
Betsy	Chrischilles						
Ryan	Carnahan						
Brian	Gryzlak						
Gi-Yung	Ryu						
Katrina	Oaklander						
Pastor	Bruce						
Brad	McDowell						
Jarrod	Field						
Abu	Mosa						
Sasha	Lawson						
Jim	McClay						
Soliman	Islam						
Vasanthi	Mandhadi						
Kim	Kimminau						
Dennis	Ridenour						
Jeff	Ordway						
Bill	Stephens						

Supplemental Online Content: Nonauthor Collaborators
*First name, last name, and suffix (if applicable) are required and will appear in PubMed.

***First Name and Middle Initial(s)**	***Last Name**	***Suffix (eg, Jr, III)**	Academic Degrees	Institution	Location (city, state/province, country)	Role or Contribution, eg, chair, principal investigator	Group (if more than 1 Group listed in the byline) and/or Subgroup (eg, Steering Committee)
Russ	Waitman						
Deandra	Cassone						
Xiaofan	Niu						
Lisa	Royse						
Vyshnavi	Paka						
Lori	Wilcox						
Janelle	Greening						
Carol	Geary						
Goutham	Viswanathan						
Jim	Svoboda						
Jim	Campbel						
Frances	(Annette)						
Haddy	Bah						
Todd	Bjorklund						
Jackson	Barlocker						
Josh	Spuh						
Louisa	Stark						
Mike	Strong						
Otolose	Fahina						
Rachel	Hess						
Jacob	Kean						
Sarah	Mumford						
Ainsley	Huffman						
Annie	Risenmay						
Olivia	Ellsmore						
Lissa	Persson						
Kayla	Torres						
Sandi	Stanford						
Mahanaz	Syed						
Rae	Schofield						
Meredith	Zozus						
Brian	Shukwit						
Matthew	Decaro						
Natalia	Heredia						

Supplemental Online Content: Nonauthor Collaborators
*First name, last name, and suffix (if applicable) are required and will appear in PubMed.

***First Name and Middle Initial(s)**	***Last Name**	***Suffix (eg, Jr, III)**	Academic Degrees	Institution	Location (city, state/province, country)	Role or Contribution, eg, chair, principal investigator	Group (if more than 1 Group listed in the byline) and/or Subgroup (eg, Steering Committee)
Charles	Miller						
Alice	Robinson						
Elmer	Bernstam						
Fatima	Ashraf						
Shiby	Antony						
Juliet	Fong						
Philip	Reeder						
Cindy	Kao						
Kate	Wilkinson						
Tracy	Greer						
Alice	Robinson						
Lindsay	Cowell						
