# Supplementary material for: Positive Airway Pressure, Mortality, and Cardiovascular Risk in Older Adults With Sleep Apnea
Source: JAMA Netw Open. 2024 Sep 11;7(9):e2432468. doi: 10.1001/jamanetworkopen.2024.32468 (PMC11391331; doi:10.1001/jamanetworkopen.2024.32468)
Supplement: Supplement 3. — Data Sharing Statement [file jamanetwopen-e2432468-s003.pdf]

Data Sharing Statement

Mazzotti. Positive Airway Pressure, Mortality, and Cardiovascular Risk in Older Adults With Sleep Apnea. *JAMA Netw Open*. Published September 11, 2024.
doi:10.1001/jamanetworkopen.2024.32468

Data
Data available: No

Additional Information
Explanation for why data not available: Data utilized in this study was accessed through the Greater Plains Collaborative (GPC) Reusable Observable Unified Study Environment (GROUSE). GROUSE is a unique de-identified data resource, created by merging Medicare and Medicaid claims with Electronic Health Records from all GPC sites. While data will not be available on public data repositories, investigators interested using the data can inquire the GPC through the GCP Front Porch (https://gpcnetwork.org/collaboration/)
